# *Hemileia vastatrix* in *Coffea* spp.: Distribution of Urediniospores Grouped by Size and Insights into Morphological Structures

**DOI:** 10.3390/jof11020109

**Published:** 2025-01-31

**Authors:** Gabriela Pelayo-Sánchez, María de Jesús Yáñez-Morales, Roney Solano-Vidal, Hilda Victoria Silva-Rojas, Dionicio Alvarado-Rosales, Simón Morales-Rodriguez, Luis Felipe Jiménez-García, Reyna Lara-Martínez, Iván Ramírez-Ramírez, Jorge M. Valdez-Carrasco

**Affiliations:** 1 Fitosanidad-Fitopatología, Colegio de Postgraduados, Campus Montecillo, Km 36.5 Carretera Federal México-Texcoco, Montecillo, Texcoco C.P. 56264, Estado de México, Mexico; gpspelayo78@gmail.com (G.P.-S.); dionicio@colpos.mx (D.A.-R.); 2 Departamento de Parasitología Agrícola, Universidad Autónoma Chapingo, Chapingo C.P. 56230, Estado de México, Mexico; roneysv@hotmail.com; 3 Producción de Semillas, Colegio de Postgraduados, Campus Montecillo, Km 36.5 Carretera Federal México-Texcoco, Montecillo, Texcoco C.P. 56264, Estado de México, Mexico; hsilva@colpos.mx; 4 Unidad de Microscopia Electrónica, Colegio de Postgraduados, Campus Montecillo, Km 36.5 Carretera Federal México-Texcoco, Montecillo, Texcoco C.P. 56264, Estado de México, Mexico; simon.morales@colpos.mx; 5 Departamento de Biología Celular, Facultad de Ciencias, Universidad Nacional Autónoma de México, Circuito Exterior, Ciudad Universitaria, Coyoacán C.P. 04510, Ciudad de México, Mexico; luisfelipe_jimenez@ciencias.unam.mx (L.F.J.-G.); rlm@ciencias.unam.mx (R.L.-M.); 6 Recursos Genéticos y Productividad, Colegio de Postgraduados, Campus Montecillo, Km 36.5 Carretera-Federal México-Texcoco, Montecillo, Texcoco C.P. 56264, Estado de México, Mexico; ivanr@colpos.mx; 7 Fitosanidad-Entomología, Colegio de Postgraduados, Campus Montecillo, Km 36.5 Carretera Federal México-Texcoco, Montecillo, Texcoco C.P. 56264, Estado de México, Mexico; jvaldez@colpos.mx

**Keywords:** anastomosis, double wall, haustoria, protuberances

## Abstract

*Hemileia vastatrix* coffee leaf rust reduces Mexican coffee production by 51%. We aimed to analyze the size and distribution of *H*. *vastatrix* urediniospores among coffee plantations, as well as the morphological structures of the uredinium. In 2015, 65 leaf samples with rust symptoms were collected from 17 coffee cultivars grown at various altitudes (229–1649 m) under different environmental conditions in 14 regions of four Mexican states. A total of 30 spores per sample were measured and grouped using the Ward centroid method, and the group distribution was analyzed. Uredinia morphology was examined via electron microscopy, and the identity of the rust was confirmed. We identified eight significant spore groups. Groups 8h and 3a had the smallest and largest spores, respectively, which were distributed in two and one state, respectively, at different altitudes. The spores in groups 1b–7f were variable within the intermediate size range, and their distribution was at least one group per state under temperate, warm, and humid conditions. The uredinium had double-cell walls in the pedicels and urediniospores, a split septum, spores with hilum and protuberances, and an oval spore shape; anastomosis was detected on vegetative hyphae and haustoria. These findings may reflect gaps in knowledge in the biological cycle of this rust.

## 1. Introduction

Mexico is known for its organic coffee production [1]. However, all Mexican coffee-producing regions, across 14 states [2], have experienced severe rust disease epidemics caused by *Hemileia vastatrix* [3], reducing coffee bean production by up to 51% [4]. In this study, we analyze some biological patterns of this rust to gain a better understanding of its behavior among coffee plantations to provide guidance for improving disease control strategies. Coffee (*Coffea* spp.; Rubiaceae) crops (perennial woody plants) are globally important, being grown in tropical and subtropical regions for the export of their seeds (coffee beans) to produce beverages. However, *H. vastatrix* limits coffee production. *Hemileia vastatrix* Berk. and Broom (Basidiomycota, Pucciniales) is the causal agent of coffee leaf rust [5,6,7]. This fungus has specific symptoms and morphological structures. On the underside of leaves, regardless of leaf age, *Hemileia vastatrix* infection causes yellow lesions that produce abundant urediniospores that aggressively infect new leaves of the same plant and of other plants. The profuse lesions result in severe host defoliation, reducing fruit production [7]. Furthermore, prolonged disease (successive years) hastens plant mortality [8].

This disease was first identified in 1869 [5] and has since become a major global problem. In Asian countries, *H. vastatrix* was detected in 1869, which caused the switch from coffee to tea crops by 1890 [9]. Over time, all countries producing coffee crops have suffered devastating rust epidemics. In the Americas, *H. vastatrix* was detected in Brazil in 1970 and in Colombia in 1983, which caused a 30% reduction in coffee yield [10,11]. This disease was also found in Guatemala [12] and Mexico [3] in 1980 and 1981, respectively, where epidemics continue to this day. The development of adequate control and mitigation measures for rust disease has relied on the study of the biological cycle of the causal agent. Such studies have focused on thallus morphology, infectious processes, host interactions (*Coffea* spp.), epidemiological characteristics, host resistance, and fungus genetic variation [5,6,7,11].

The success of *H. vastatrix* in causing devastating epidemics is linked to its physiological races [13], that reduce host resistance [10]: 50 races have been characterized [14], and Race II is the most widespread [10]. The genetic diversity of this pathogen is high, according to a haplotype analysis: 68 and 92 haplotypes have been characterized in Peru [15] and Brazil [16], respectively. The races present in Mexico are unknown, because no differential sets of hosts are available (unpublished data). This knowledge gap precludes the consideration of cultivars with horizontal rust resistance among coffee breeders. As a result, both traditional and highly susceptible coffee varieties have been replaced with vertically resistant varieties to control this rust in Mexico [17].

Patterns of phenotypic variation (in addition to genetic variation) have been reported for *H. vastatrix*. For example, environmental conditions can regulate pathogenesis: lesions with larger amounts of sporulation are induced at 27 °C (day) and 22 °C (night) and under high light intensity and low levels of nitrogen fertilization [18]. Pronounced variations were recorded in urediniospore germination and germ tube elongation among 10 *H. vastatrix* populations across a range of temperatures (15, 20, 25, and 30 °C) [19]. The teliospores of *H. vastatrix* vary in both the size and shape of their basidiospores during the winter in Brazil [20]. Furthermore, molecular groups and subgroups have been characterized in *H. vastatrix* populations in relationship “to host adaptation” among diploid and tetraploid coffee species in various locations [12,21,22].

The *H. vastatrix* population can be genetically disturbed by annual temperature and rainfall fluctuations [22]; e.g., epidemics in Colombia and Central America were related to recurrent decreases in daytime temperatures [12]. Climatic changes (higher temperatures and low rainfall amounts or drought) are altering the physiology of coffee plants which, when coupled with the lack of adequate fertilization, have contributed to coffee plant stress in Mexican orchards.

In Mexico, coffee fields are planted with different cultivars under different environmental conditions; *H. vastatrix* spore production is abundant, and spore dispersion is continuous. Considerable efforts have focused on controlling this rust disease, as the application of synthetic chemical fungicides hinders or prevents Mexican coffee from being considered organic [1,23]. Some of these controls measured have included diminished or entirely removed shading of coffee plantations [24], the use of biocontrol agents to mycoparasitize *H. vastatrix* urediniospores [25], genetically reinforcing coffee varieties, and changes to cultural practices [26].

Given the complexity of this disease, in this study, we aim to answer the following question: Is *H. vastatrix* leaf rust disease related to regional coffee plantation conditions or to the use of unknown morphological structures in addition to its known structures to escape control measures? As coffee leaf rust is a devastating disease of coffee plants, and given the lack of permanent control measures, we devised two hypotheses: (1) the size of the urediniospores of *H. vastatrix* differs among coffee plantation regions with different cultivars and environmental conditions (altitude, temperature, and humidity), and (2) some of the morphological structures of *H. vastatrix* have not yet been described.

Thus, the aims of this study are to analyze the distribution of *H. vastatrix* urediniospores, grouped by size, to comprehensively examine the behavior of this rust among coffee plantations, and to re-examine and describe the morphological structures involved during urediniospore development, as well as other structures, such as the appressoria, haustoria, and hyphae, outside the surfaces of infected leaves and within tissues. This study deepens our understanding of rust biology; the findings can be used to develop and improve alternative rust disease management strategies.

## 2. Materials and Methods

### 2.1. Distribution of Urediniospores

#### 2.1.1. Symptomatic Leaf Samples and Location Characteristics

Several coffee cultivars with symptomatic leaves indicating *H. vastatrix* infection were collected from different regions, altitudes, and environments. The urediniospores from the uredinia were analyzed. We obtained samples from 17 cultivars (Bourbon, Blue Mountain, Catuai, Caturra Amarillo, Caturra Rojo, Colombia, Costa Rica 95, Garnica, Maragogipe, Mundo Novo, Moka, Oro Azteca, Pacamara, Pluma Hidalgo, Robusta, Surinam, and Typica) of *Coffea* spp. (*Coffea arabica*, n = 13; *Coffea canephora*, n = 1; and Catimores, n = 3 (Timor hybrid × Caturra Rojo)) from August to November 2015 from 14 regions of 11 coffee-producing municipalities in 4 Mexican states: Chiapas, Veracruz, Oaxaca, and Puebla (ordered according to coffee production volume) [27]. The sample sites ranged from 229 to 1649 m above sea level (m a.s.l), and data regarding environmental conditions (climate condition, rainfall, and temperature) were retrieved from online databases [28,29] (Table A1 and Table A2). In each of the 14 sampling regions along each cardinal direction, 4 fully expanded symptomatic leaves in full sun (1 leaf for each cardinal direction) were collected from each of 4 randomly selected trees (distance around tree was approximately 3 m) of a given cultivar with rust disease symptoms (n = 3808 leaves collected, 17 cultivars × 14 regions × 4 trees × 4 cardinal directions).

#### 2.1.2. Sample Subsets

Symptomatic leaves with rust lesions were screened under a stereo microscope (Nikon, mod SMZ800, Tokyo, Japan) to ensure that the uredinium and urediniospores in the lesions during each sampling were in the same apparent stage. We selected leaves with lesions free of mycelia or conidia from other fungi and with abundant *H. vastatrix* urediniospores. After this screening, 265 leaves remained. A subset of 65 leaves was finally selected, after a second screening for rust lesions with the brightest yellow color and abundant urediniospores.

### 2.2. Urediniospore Size

#### Size

The urediniospore size (µm) of the 65 selected leaf samples was measured under a stereo microscope at high magnification (30–63 times). Spores were removed from the uredinium using a fine-point needle (Nipro syringe, mod. 29G × ½″, Jawa Barat, Indonesia). The spores were then mounted in 85% lactic acid (Reasol, Mexico City, Mexico). For each leaf, 30 urediniospores (30 × 65 leaves = 1950 spores) were measured under a light microscope (100×) (Nikon^®^ Eclipse E400, Tokyo, Japan). Additionally, the spore germination of some samples was observed via light microscopy. The spores were immersed in drops of sterile water at room temperature for 48 and 72 h, and montages with cotton-blue color were observed.

### 2.3. Grouping of Urediniospores by Size

#### 2.3.1. Size Range

Urediniospore width, length, and wall thickness were recorded. Their corresponding size ranges (µm) were calculated with a 95% interval range (from the 30 urediniospores measured), as well as the minimum and maximum values (each with 2.5% intervals from the remaining urediniospores) [30] (Table A1).

#### 2.3.2. Grouping

The urediniospore measurement data from 65 leaves (one lesion per leaf) (Table A1) were analyzed using analysis of variance (ANOVA) and Duncan’s multiple range test (*p* ≤ 0.05) via Statistical Analysis Software (SAS version 9.0; SAS Institute Inc., Cary, NC, USA) to group the urediniospores by size range. The between-mean differences were assessed. The mean width and length were transformed into a new set of uncorrelated variables via principal component (PC) analysis [31] to determine the components (Print1 and Print2) that most accurately described the variables (urediniospore size and shape). Cluster analysis was performed using the Ward grouping-by-centroid method (separation distance of 0.34) [32] to distinguish similar groups within the subsets. Groups with the maximum variation between the groups but with minimum variation within the group were determined using ANOVA. The overall urediniospore size ranges were determined into subsets within each group (mixed oval and reniform shapes) (Table 1) according to the morphological reniform and oval shape sizes (Table 2), and the corresponding mean and standard deviation values.

### 2.4. Distribution of Groups

The number of samples and the origin of collection of each of the samples were analyzed for each group to determine the distribution of the spore size among the states, coffee cultivars, and the degree of host resistance or susceptibility [17]. The location altitude and environmental conditions (temperature and humidity) were recorded (Table A1 and Table A2).

### 2.5. Descriptive Insights into Morphological Structures

#### 2.5.1. Re-Examination of Uredinia and Urediniospores

Four methods were used to perform a high-magnification search (1000 times using a light microscope; 1000–20,000 times using an Electron Microscope) for previously undescribed structures.

##### Hand-Prepared Slide Montages

Spores and other related morphological structures (uredinium thallus) were re-analyzed on slide montages under a light microscope. We compared these montages with those previously prepared for the measurement of the size range of urediniospores. The montages were prepared via the vertical sectioning of the uredinium sori from the leaves with lesions using a thin razor blade (Gillette^®^, Naucalpan de Juarez, Estado de Mexico, Mexico). This was followed by mounting the tissue sections with superficial and immersed structures.

##### Histological Analysis

Montages were prepared for the light microscopy examination of the internal cells of symptomatic leaf tissue and the inter- and intracellular structures (e.g., the hyphae and haustoria) of the leaf rust lesions with uredinia. Leaf fragments (n = 15; approximately 1.0 cm^2^) immersed in paraffin wax (Paraplast^®^-Sigma-Aldrich, St. Louis, MO, USA) were vertically sectioned (10 µm thick) using a rotary microtome (Leica Company Mod. RM2125, Xi’an, China). Histological sections (n = 80) were stained with fast safranin-green (Sigma-Aldrich, St. Louis, MO, USA), dissolved in methyl cellosolve (Sigma-Aldrich, St. Louis, MO, USA), and analyzed under a light microscope at high magnification (100×) [35].

##### Scanning Electron Microscopy (SEM)

SEM (JSM-6390/LGS; Jeol, Tokyo, Japan) was performed to more closely examine the uredinium structures. Typical rust lesions with abundant uredinia structures (e.g., featuring urediniospores, pedicels, and sporophores) on 3–5 leaves from the Caturra Rojo (highly susceptible) cultivar were selected and cut into square pieces (5 × 5 mm). The lesion pieces were grouped into montages and observed via SEM at 10 Kv [36]. The representative structures of the urediniospores, hyphae, pedicels, sporophores, and conidiogenous cells with atypical morphologies were measured and photographed. Additionally, external atypical superficial hyphae among the uredinium from germinated urediniospores, as well as the internal hyphae and haustoria in infected tissue, were examined via SEM.

##### Transmission Electron Microscopy (TEM)

The internal composition of the urediniospore cell wall was examined using TEM (JEM-1010, JEOL, Tokyo, Japan). Spores were fixed in situ (3% glutaraldehyde in 0.1 M phosphate buffer, pH = 7.2), immersed in London white resin (LR) (agar scientific; Sigma-Aldrich, Merck, Darmstadt, Germany) for TEM, vertically sectioned (50 and 60 nm in thickness), mounted, and observed at 80 Kv [37].

### 2.6. Species Corroboration

*H. vastatrix* taxonomy was confirmed via morphology [5,38] and coffee leaf symptomatology [7]. For the characterization via molecular analysis, DNA was extracted from 16 randomly selected subsamples (Table A1), and PCR (Phire Plant Direct PCR Kit; Thermo Scientific^®^, Plainville, MA, USA) was conducted using the internal transcribed spacer regions (ITS) of rRNA genes and ITS5 and ITS4 primers [39], following previously described thermocycling conditions [40] (Appendix B). The edited sequences were analyzed using BLASTn (Pre-formatted databases for BLAST nucleotide) and submitted to the NCBI GenBank database (https://ftp.ncbi.nlm.nih.gov/blast/db/, accessed on 18 November 2024).

## 3. Results

### 3.1. Urediniospore Size Range Analysis

**Figure 1:**
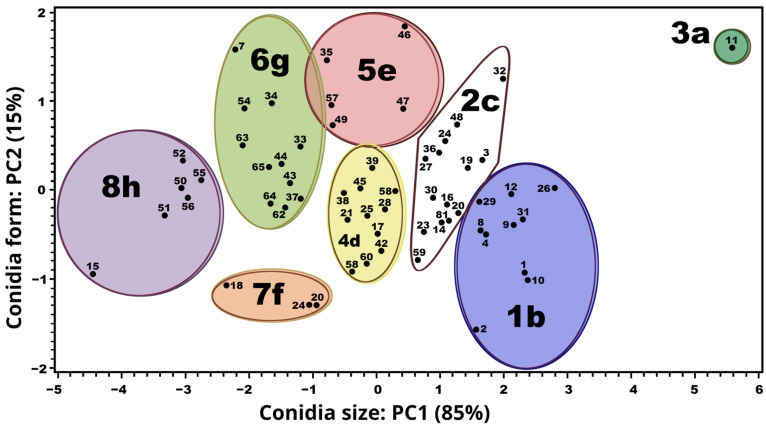
Eight groups (1–8) of urediniospore samples collected from 65 leaves, grouped by size (µm), and analyzed using the Ward centroid method. Different letters (a–h) indicate significant differences between groups (*p* < 0.0001); PC = principal component.

The PC (principal component) analysis of the urediniospore size range and shape generated two linear combinations from the 65-sample subset of leaves (Table A1): PC1 (Prin1) = 0.797 length + 0.603 width, and PC2 (Prin2) = −0.603 length + 0.797 width. PC1 explained 85% of the total variance in the urediniospore size range and comprised eight significantly distinct groups, 3a–8h (*p* < 0.0001) (Figure 1; Table 1). PC2 explained the remaining 15% of the variance in the urediniospore shape (reniform and oval) (Table 2).

### 3.2. Urediniospore Groups and Distribution

#### 3.2.1. Groups

The urediniospore size range of the eight groups of urediniospores (3a, 1b, 2c, 4d, 5e, 7f, 6g, and 8h) from the 65-sample leaf subset was significantly different (*p* < 0.0001 (Figure 1; Table 1)). The samples in the groups were obtained from different states, and the numbers of samples in each group differed. Groups 2c and 6g were the most frequently sampled (eight times each). Samples from group 2c were from Puebla, Oaxaca, and Chiapas; group 6g samples were from Puebla, Chiapas, and Veracruz. Group 2c samples were most frequently sampled (four times) from Chiapas. Groups 1b and 4d contained seven and six samples, respectively: the former were obtained from Puebla, Oaxaca, and Veracruz; the latter were from Oaxaca, Chiapas, and Veracruz. Groups 5e, 7f, and 8h contained fewer with three, three, and two samples, respectively. Group 5e samples were from Chiapas and Veracruz; group 7f samples were from Puebla, Oaxaca, and Chiapas; and group 8h samples were from Oaxaca and Veracruz. Group 3a contained only one sample from Puebla. The cultivars (one to seven by distribution group) and altitudes (229 to 1649 m o.s.l.) of the samples in each group were diverse and are provided in Table 1; the locations (regions) are listed in Table A2.

#### 3.2.2. Distribution

Group 8h (Figure 1) (Table 1) was distributed in two regions in two states, in six susceptible cultivars at medium and high altitudes (Table 1) under mild–warm and warm temperatures and humid conditions (Table A1 and Table A2). In contrast, group 3a (Figure 1) (Table 1) was distributed in one region in one state for one highly susceptible cultivar at the lowest altitude and under warm, wet, and humid conditions (Table A1 and Table A2).

The urediniospores of the other six groups (Figure 1) were in the intermediate size range (Table 1). Group 1b was mainly distributed in four regions in one state, and found in five cultivars (two highly susceptible, one susceptible, and two resistant) at low to high altitudes under mild–warm to warm temperatures, as well as wet and humid conditions. Group 2c was mainly distributed in four regions in one state in three cultivars (one highly susceptible, one susceptible, and one resistant) at medium and high altitudes under temperate, mild–warm to warm, wet, and humid conditions. Group 4d was distributed in three regions in one state in three susceptible cultivars (one highly susceptible and two susceptible) at medium to high altitudes and under mainly temperate, warm temperature, and humid conditions. Group 5e was mainly distributed in two regions in one state in two cultivars (one highly susceptible and one susceptible) at high altitudes under temperate conditions with mild–warm temperatures as well as wet and humid conditions. Group 6g was mainly and equally distributed in three regions in two states: in the one state, the samples were from two cultivars that were highly susceptible; in the second state, the samples were from four cultivars (three susceptible and one resistant) at medium to high altitudes under mainly temperate, mild–warm to warm temperatures, and humid conditions. Group 7f was distributed in three states (only once per state) in two cultivars (one highly susceptible and one resistant) at medium and high altitudes under mild–warm to warm temperatures as well as wet and humid conditions (Table 1 and Table 2; Table A1 and Table A2).

### 3.3. Re-Examined Morphological Structures of Uredinia

#### 3.3.1. Hand-Prepared Slide Montages

**Figure 2:**
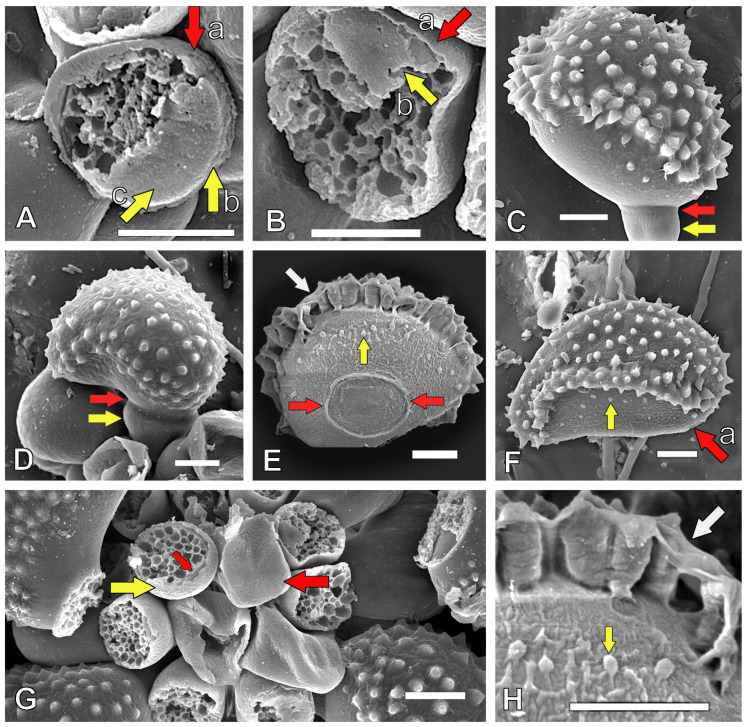
Description of morphological features of *Hemileia vastatrix*. (**A**,**B**) Pedicel double wall: (a) external wall (red arrow); (**A**(b)) internal wall (yellow arrow); (**A**(c),**B**(b)) internal wall remnants on apical pedicel (yellow arrows). (**C**,**D**) Split septum (red arrow) and pedicel (yellow arrow). (**E**) Hilum or mark in oval spore (between red arrows). (**E**,**F**,**H**) Ventral spore protuberances (yellow arrow). (**E**,**H**) Mucilage-like remnants (white arrow) over marginal echinulations. (**F**) Hilum in reniform spores (red arrow pointing to flat mark). (**G**) Pedicels; right: entire apical internal wall; left: partial internal wall (red arrows). (**H**) Magnified view of ventral spore protuberances (yellow arrow), marginal echinulation, and mucilage-like remnants (white arrow). Images were generated via scanning electron microscopy. Scale bar in Figure 2A is 3 µm, and all other scale bars are 5 µm.

**Figure A1:**
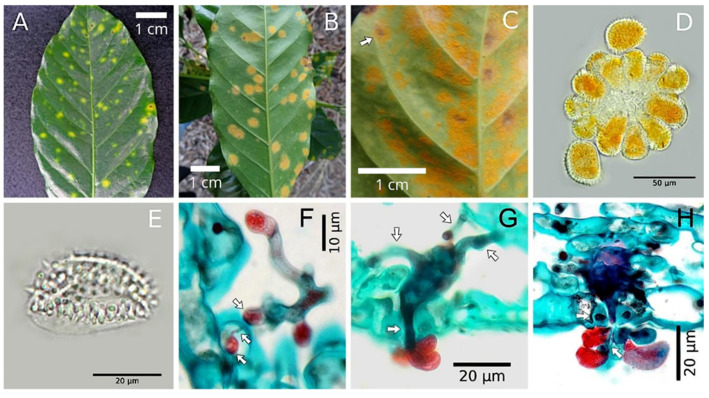
Leaves of *Coffea* sp. with symptoms of coffee leaf rust disease and infectious structures of *Hemileia vastatrix*. (**A**) Leaf surface showing rounded, oval, and chlorotic to yellow lesions; (**B**) leaf underside showing individual lesions and superficial uredinia of orange urediniospores; (**C**) leaf underside showing advanced disease symptoms and coalescent orange lesions with necrotized center; (**D**) uredinium with hyaline-walled urediniospores and orange cytoplasm; (**E**) reniform urediniospore hyaline; (**F**) intercellular hyphae with short branches and expanded terminations, and intracellular haustorium (white arrow) with neck (hyphae filament) (white arrow) in cytoplasm of cell in spongy parenchyma. Interior and exterior of stoma: (**G**) uredinium with immature and superficial urediniospores and three hyphae (up) forming protosorus; (**H**) uredinium with initial and advanced development of urediniospores. (**D**–**H**) are light microscopy images.

The symptomatic leaves hosted *H. vastatrix* uredinia and urediniospores with typical morphologies (Figure A1A–E), as previously reported. However, some oval urediniospores were noted (Table 2; Figure 2C,E) which were larger than the reniform spores.

#### 3.3.2. Histological Analysis

The histological analysis revealed the details of the structures of the rust’s intercellular hyphae and thalli (Figure A1F–H), which have also been previously reported.

#### 3.3.3. Scanning Electron Microscopy (SEM)

**Figure 3:**
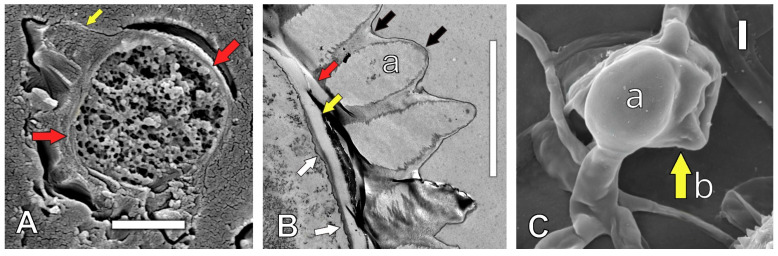
Double wall of urediniospore. (**A**) Vertical section of urediniospore at low magnification: cell wall (red arrows) and echinulations (yellow arrow). (**B)** Double wall of urediniospore at high magnification: an external wall (red arrow) with echinulation (a) and surface matrix-like mucilage (black arrows); an internal wall (yellow arrow) over a basal plasma membrane (white arrows). (**C**) Appressorium (a) with matrix substance and (b) a substance resembling mucilage (yellow arrow). (**A**,**B**) Transmission electron microscopy images; (**C**) scanning electron microscopy image. Scale bars are 5 µm.

Five morphological structures were identified (Figure 2): (a) We observed a double wall in the urediniospore pedicel: an external wall (Figure 2A(a),B(a)), an internal wall (Figure 2A(b), and internal wall remnants on the apical pedicel (Figure 2A(c),2B(b)). (b) We observed a split septum delimiting the young (Figure 2C) and mature (Figure 2D) urediniospores and the pedicel. (c) We noted a spore hilum or mark (an attachment area between the spore and the pedicel remaining after the split septum breaks down). The hilum (Figure 2E) was detected toward the middle of the ventral side in the oval urediniospores; in contrast, the hilum was located at the extremity of the ventral basal side in the reniform spores (Figure 2F(a); lateral view), where the pedicel attached early (Figure 2D). (d) We identified an entire apical and internal wall as a result of the half-cross wall at the septum on the pedicel (Figure 2G (right)) (after the split septum breaks down); we also noted internal wall remnants from the apical pedicel (Figure 2G(left),A(c),B(b)). (e) Protuberances (yellow arrows) were observed (Figure 2H). The ventral sides of the spores were not smooth in some development stages; the oval urediniospores had abundant protuberances (Figure 2E,H; close view); fewer protuberances were detected on the reniform urediniospores (Figure 2F). (f) In addition, a matrix that appeared to contain a mucilaginous substance was observed, covering the top of (Figure 2E,H; close view) and between the echinulate ornamentations (Figure 3B(a)). (g) Moreover, the appressoria (Figure 3C(a)) were covered with a mucilaginous matrix (Figure 3C(a,b)); this substance on this structure has not been previously reported.

#### 3.3.4. Transmission Electron Microscopy (TEM)

(h) A double spore wall was observed. A urediniospore structure was identified on the spore wall (Figure 3A): a urediniospore double cell wall (Figure 3B, in a vertically sectioned urediniospore): an external cell wall, from which echuinulate ornamentations arose (Figure 3B(a)) (also covered by a matrix of a mucilaginous substance); and an internal wall, which was positioned adjacent to the external wall (yellow) and over the plasma or cell membrane (white arrow).

### 3.4. Other Analyzed Structures

#### 3.4.1. Hyphal Anastomoses

**Figure 4:**
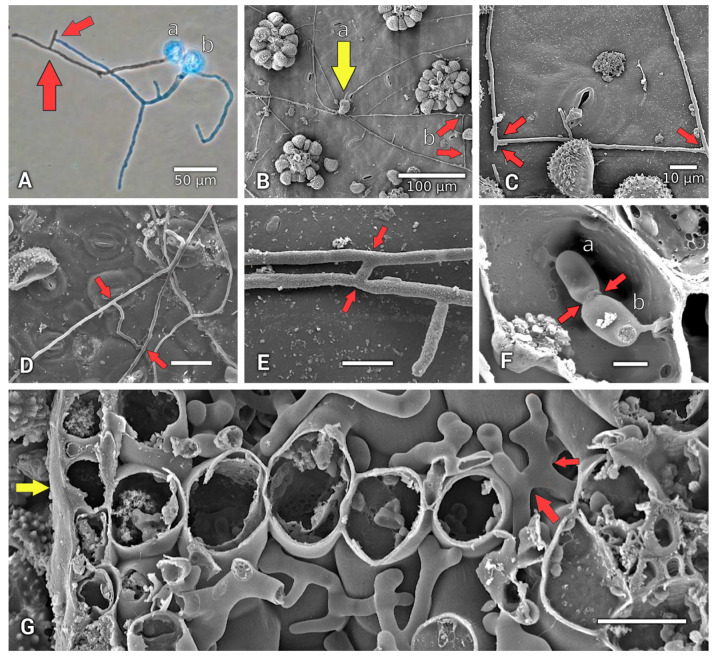
Hyphae and haustoria anastomoses of *Hemileia vastatrix*. (**A**) Hyphae anastomosed (red arrows) between two germinated urediniospores (a,b). (**B**) Anastomosed hyphae (b; red arrows) from one germinated urediniospore (yellow arrow) on coffee leaf surface. (**C**) Magnified view of same anastomosed hyphae (red arrows). (**D**,**E**) Leaf surfaces with anastomosed hyphae; long and short bridges (red arrows) from germinated urediniospores. (**F**) Intracellular anastomosed haustoria (red arrows). (**G**) Intercellular (sponge parenchyma) anastomosed hyphae (red arrows) and vertical leaf cross-section (yellow arrow). (**A**), light microscopy image (40×); (**B**–**G**), images from a scanning electron microscope (SEM); (**F**,**G**), histological sections under SEM. (**D**,**G**), Scale bars = 20 µm; (**E**,**F**), Scale bars = 5 µm.

Two additional structures were documented. (i) Four types of hyphal anastomoses, defined as fusions of hyphae and other fungal structures, were noted (Figure 4A,B,D,E): (1) in vitro from two germinated urediniospores (Figure 4A) between the branched hyphae of germ tubes (Figure 4A(a,b)) and (2) in situ on the coffee leaf surface among the uredinium of one germinated urediniospore (Figure 4B(a)) between branched hyphae (Figure 4B(b)); a close-up view is presented in Figure 4C. Moreover, hyphal anastomoses were observed between hyphae with long (Figure 4D) and short (Figure 4E) branched hyphae bridges from germinated urediniospores; (3) anastomosed intercellular hyphae were observed (Figure 4G) in infected tissue in situ, forming a network among the sponge parenchyma of the coffee leaf tissue.

#### 3.4.2. Haustoria Anastomoses

(4) We observed haustoria anastomoses in infected tissue (in situ) between two intracellular haustoria (Figure 4F(a,b)).

### 3.5. Hemileia vastatrix Confirmation

The identity of this species was verified using its morphology (Figure 2; Appendix B), urediniospore size (Table 1 and Table 2), symptomatology (Figure A1) (material deposited in Colegio de Postgraduados Mycology Plant Disease Herbarium (CMPH); Table A1), and molecular analyses. The 16 *H. vastatrix* sequences generated in this study were confirmed using BLASTn (Table A1; Appendix A) and deposited in GenBank (Acc. no. KX260246–KX260253, MF417744–MF417745, MF417747–MF417748, MF417750–MF417751, and OL700032–OL700033). The phylogenetic details of these 16 *H. vastatrix* sequences aligned with those of the *H. vastatrix* sequences from Colombia (Accession Nos. EF394119, EF394120, EF394122, and EF394129) and Brazil (Accession Nos. MF627758 and MF627827).

## 4. Discussion

### 4.1. Groups of Urediniospores and Distribution

Our first hypothesis was confirmed: *H. vastatrix* urediniospores grouped by size exhibit distinct distributions among coffee plantation regions. The eight urediniospore size groups in this study were obtained from only 65 representative leaves from 14 coffee plantation locations. However, all leaves were subjected to a rigorous selection process to ensure consistent quality standards among the leaves. Collecting more samples would likely lead to the identification of more groups in the *H. vastatrix* population. The results of molecular analysis (21) indicate a C3 group and three subgroups in a *H. vastatrix* population, indicating host adaptation (*Coffea* sp.) and wide distribution [12,21,22]. As such, the groups identified in this study could represent the phenotypic expression of an unknown molecular group.

Some groups were not found in all locations. For example, group 1b was not found in Chiapas, groups 2c and 7f were not found in Veracruz, group 6g was not obtained from Oaxaca, and group 4d samples were not from Puebla. Group 8h was found only in Oaxaca and Veracruz, group 5e in Chiapas and Veracruz, and group 3a in Puebla. Studies are required to determine the relationships among the groups, locations, coffee cultivars, and environmental conditions. Instead of searching for morphological groups, as in this study, most studies of pathogenic fungi have focused on changes in morphogenesis to maintain or increase virulence in response to external factors [41], on qualitatively explaining morphological differentiation during the pathogenic life cycle [41], and on searching for ecotypes [42] or races [14] in *H. vastatrix*.

The size range of urediniospores may be associated with spore age because of the sympodial formation of urediniospores on sporogenous cells [6,34,43], although other factors should be considered (e.g., environmental conditions and/or host adjustment). For instance, independent studies in geographically diverse areas and conducted in different years [6,7,33,34], which we comparatively analyzed (Table 2), have reported varying *H. vastatrix* urediniospore size ranges (Table 2), e.g., differing in extreme size or in the minimum and maximum width and length. However, in addition to spore age, the differences in size range may be because of the urediniospore populations in previous studies [6,7,33,34] being from different groups or due to differences in the methodology and equipment used. The role of these spores (variations in size range) in pathogenicity should be analyzed.

*H. vastatrix* can be genetically disturbed by annual temperature and rainfall fluctuations [22]. Epidemics in Colombia and Central America were related to recurrent decreases in daytime temperatures [12]. Therefore, we suggest that the continuous coffee leaf rust epidemics in Mexican coffee plantations over the last 42 years, since rust disease was first identified in 1981 [3], have been due to environmental as well as other factors. The temperature widely fluctuated during the day and night in three of the sampled states (Table A2). Additionally, the urediniospore groups were distributed at low and high altitudes, in temperate, mild–warm, and warm climates, and across a broad range of rainfall conditions (Table A2), all of which favor the spread of leaf rust. Another factor in these epidemics is the *Coffea* spp. cultivars. Evidence shows that *H. vastatrix* is slowly morphologically adapting to its coffee hosts, presumably to maintain its fitness [22]. In the studied regions, cultivars Costa Rica 95, Oro Azteca, and Robusta were initially planted as *H. vastatrix*-resistant cultivars [17], but they are now susceptible to infection in some regions in Chiapas, Oaxaca, Puebla, and Veracruz (unpublished data). Consequently, the Caturra Amarillo, Caturra Rojo, and Pluma Hidalgo cultivars, as well as other highly susceptible cultivars, have been replaced with new resistant cultivars. However, these cultivars have also begun to show signs of the disease [17].

### 4.2. Morphological Structures 

Our second hypothesis, which stated that *H. vastatrix* has several hidden and undescribed structures, was supported. The detected split septum, spore hilum, and internal wall on the apical surface of the pedicel (Figure 2C–E,G (right)) indicate that these structures allow spore secession, as reported for other fungus species *(Sympodiophora*) [44]. These findings indicate that, once a spore matures, the external wall of the split septum ruptures, and the internal walls longitudinally divide (or a double septum on the internal wall forms during sporogenesis) [44] (Figure 3.51, p. 39), releasing the spore. Thus, our study suggests that *H. vastatrix* undergoes schizolytic urediniospore secession, as previously reported for different fungal genera [44,45]. *H. vastatrix* schizolytic spore secession enables simple mechanistic spore release from the pedicel, and epidemiologically increases disease spread. This contrasts with spores reliant on rhexolytic secession [44,45], where a particular cell forms below the spore. This implies that, in addition to requiring more time to complete rhexolytic secession, extra energy is required, and the lateral walls only undergo lysis for spore secession at a later stage. Moreover, we found that the pedicel and urediniospore had double walls (Figure 2A,B,G and Figure 3B). We inferred that this double wall contributes to *H. vastatrix* spore formation through enabling spore secession. Based on these findings, we propose that the inner wall functions to protect the spore cytoplasm from adverse environmental conditions. In addition, the outer wall may provide defense against toxic secretions of the coffee plants as well as chemical applications. Fungicides applied to control *H. vastatrix* mainly acted on the germ tubes of germinated urediniospores [6]. Double spore walls are also present in *Gymnosporangium juniperi*-*virginianae* (apple rust), where the basidiospore ontogeny is holoblastic, and the basidiospore double wall is a continuation of the sterigma double wall [43]. The sporogenous cells of *Puccinia coronata* f. *avenae* and teliospores of *Cronartium ribicola* also have double cell walls, although the spore bud and teliospore germ tube, respectively, emerge only in the inner cell wall, as the outer sporogenous cell wall is broken during the process [43]. Some rust species have only one wall, e.g., *Melampsora lini* has a single wall covered by a pellicle (7–20 nm thick), where the wall comprises three layers that differ in appearance [43]. A cell membrane (a figure of this positioning for a cell membrane was reported in 1970 in an Oomycota [46] (Figure 3.2; p. 50)) behind the two cell walls was also documented in this study. Thus, rust spore walls are complex, and their spore protection function is an inference. Additionally, the mucilaginous matrix present on the *H. vastatrix* spore surface may have several functions in urediniospore protection, among others [47]. For example, the mucilaginous matrix could assist with preventing spore dehydration, facilitating adherence to the leaf surface, or repelling fungicides while allowing spore germination and host infection. Such effects can be achieved because of the high density of the mucilage, with its oil conferring hydrophobicity [47]. The incorporation of an adherent in fungal control substances should prevent surface tension, allowing the direct contact of the control substance with the cell wall and preventing spore escape from such control measures. A mucilaginous matrix covering mature *H. vastatrix* sori was reported in 1983 [6] (Plate 32; Figures 64,65; p. 120); a similar mucilage-like material was observed for the first time on appressoria in this study (Figure 3C). From an epidemiological perspective, this extra urediniospore substance indicates that coffee leaf rust spores can easily attach to the underside of the host leaf surface; as such, spores are able to cause infection because of this mucilage-like hydrated material under low relative humidity. In general, the mucilage protects fungal conidia from adverse environmental conditions, strengthening pathogen infection, as shown for *Hirsutella satumaensis* (an insect pathogen) [47]. Further studies are warranted on the mucilage on *H. vastatrix* structures to control coffee rust. Furthermore, our finding that the ventral sides of urediniospores have protuberances suggests that, once the spores are released, the protuberances have a strong ability to adhere to the host surface, thus elevating the probability of infection. Based on these structural insights, we hypothesize that *H. vastatrix* has several morphological features that increase its epidemiological efficacy.

### 4.3. Insights into Other Features of Infected Tissue

Appressoria, intercellular hyphae, and haustoria structures per se, were previously reported [5]. Additionally, the observed anastomoses of the hyphae (Figure 4A–E,G) (via hyphal bridges) and haustoria (Figure 4F) (red arrows, Figure 4) indicate the generation of compatible nuclei to form the dikaryon [48] in *H. vastatrix*’s vegetative cellular structures. Anastomosis has been reported in several rust species, e.g., soybean rust *Phakopsora pachryizi* [49], which shows anastomoses of the germ hyphae [49] (In: Figure 1(a); p. 165), although anastomoses have not previously been documented for *H. vastatrix*. Dikaryotic nuclei in all (asexual and sexual) cells of the *H. vastatrix* thallus have been reported [50]. The anastomose condition has not been observed or mentioned for *H. vastatrix* [50]; therefore, this study is the first to document anastomosis in *H. vastatrix*. Thus, future studies could examine if *H. vastatrix* is gaining compatible nuclei through anastomosis to result in somatic hybridization [51] via mitosis [48]; this is one possible factor, among others, that could allow *H. vastatrix* to accumulate genetic variation [51].

The genetic variation in *H. vastatrix* is evident from the constant increases in the number of races [13], haplotypes [16], linage C3 group, and subgroups [22]. This genetic variation has been identified through molecular analyses (amplified fragment length polymorphism, internal transcribed spacer polymorphism, microsatellite-primed PCR, random amplified polymorphic DNA, restriction fragment length polymorphism, and single-nucleotide polymorphism) [12,22,52,53,54]. The attributes of *H. vastatrix* described in this study can be studied to improve crop management practices.

## 5. Conclusions

To the best our knowledge, this is the first study to describe the presence and distribution of *H. vastatrix* urediniospores grouped by size in various Mexican regions in different coffee cultivars and under various environmental conditions. In addition, we provide new insights into the double cell wall of the urediniospores and pedicel and the mechanism of spore release, as well as report an oval urediniospore shape, protuberances in the ventral side of spores, an appressorium with a matrix substance, and anastomoses of the hyphae and appressoria. This study improves our understanding of the variations in the life cycle of the coffee rust disease pathogen across regions. Our findings can be used to strengthen and/or devise control strategies and efforts to mitigate or halt future coffee rust epidemics while pursuing sustainable coffee production.

## Figures and Tables

**Table 1:** Characteristics of each group at the collection sites, number of samples, and overall sizes of urediniospores with mixed oval and reniform spore shapes.

Group ^1^	Leaves ^2^ per Group	Number of States Sampled	Altitude Range (m)	Number of Cultivars (Cultivar Name)	Spore Size (µm) Range (Width ^3^ × Length ^4^) ^5^
3 ^1^ a ^6^	1	1	229	1 (Caturra Rojo)	29.5–40 ^3^ × 19–32.5 ^4^ (–35) ^5^ a ^6^
1b	11	3	229–1100	5 (Catuai, Caturra Rojo, Costa Rica 95, Oro Azteca, Pluma Hidalgo)	23–38 × 15–29 (–37) b
2c	16	4	350–1649	7 (Bourbon, Catuai, Caturra Rojo, Oro Azteca, Pacamara, Pluma Hidalgo, Robusta)	(20–) ^5^ 23–37.5 (–39) × (12.5–) 13–28 (–30)c
4d	12	4	389–1318	7 (Bourbon, Caturra Rojo, Maragogipe, Oro Azteca, Pacamara, Pluma Hidalgo, Robusta)	(20–) 24–36 (–38) × (14–) 15–25 (–28) d
5e	5	2	1241–1649	4 (Bourbon, Blue Mountain, Caturra Rojo, Garnica)	(21–) 25–35 × (14.5–) 16–27 (–30) e
7f	3	3	559–653	2 (Caturra Rojo, Robusta)	(24–) 28–36 (–38) × 14–25 (–26.5) f
6g	11	3	389–1332	7 (Caturra Amarillo, Colombia, Caturra Rojo, Costa Rica 95, Mundo Novo, Oro Azteca, Typica)	(20–) 23–35 (–37) × 14–27 (–28) g
8h	6	2	773–1318	6 (Blue Mountain, Garnica, Moka, Pacamara, Pluma Hidalgo, Surinam)	(19–) 22–35 × 14–26 h
Overall spore size (all 8 groups): Average: width (±standard deviation (SD)) × length (±SD):					(19–) 22–40 × (12.5) 13–32.5 (–37) 30.56 (±3.32) × 20.96 (±3.04)

^1^ Groups determined by centroid method analysis (separation distance, 0.34); ^2^ total number: n = 65 leaves (Table A1); ^3,4^ determined with 95% interval (range) [30]; ^5^ numbers in parenthesis are extreme spore values (2.5% interval: (minimum–); (–maximum)); ^6^ different letters indicate significant differences between groups (*p* < 0.0001).

**Table 2:** Comparison of *Hemileia vastatrix* urediniospore shape and size.

Spore	Spore Size (µm) Range ^1^ (Width ^2^ × Length ^3^)	Wall Thickness	Average (±Standard Deviation)
Shape and size (from this study) ^4^:			
Reniform	^1^ (18–) 22–37 ^2^ (–40) × (15–) 17–29 ^3^	1.0	30.50 ^2^ (±2.77) × 26.32 ^3^ (±2.69)
Oval	(27–) 29.5–37 (–39) × (24) 27–30 (–35)	1.0	34.03 (±3.36) × 26.93 (±4.18)
Overall spore size ^5,6^:	(19–) 22–40 × (12.5–) 13–32.5 (–37)	1.0	30.56 (±3.32) × 20.96 (±3.04)
Comparative Size (from the literature) ^6^:			
[6] ^7^	(20.5–) 25–40 (–43) × (10.2–) 15–32	1.0–1.5	
[7]	25–35 × 12–28	- ^8^	
[33]	26–40 × 18–28	1.0–2.0	
[34]	28–36 × 18–28	1.0	

^1^ Numbers in parenthesis are extreme spore width and length values (2.5% interval: (minimum–), and (–maximum)) [30]; ^2,3^ 95% interval range [30]; ^4^ urediniospore size (µm): 30 urediniospores were measured for each morphological shape; ^5^ from all 8 groups described in Table 1; ^6^ including reniform and oval shapes; ^7^ measurements derived from meta-analysis of 10 articles [6]; ^8^ data not available.

## Data Availability

Colegio de Postgraduados–Campus Montecillo–México; at: https://www.colpos.mx/cp/campus-montecillo, accessed on 3 January 2025.

## Appendix A

**Table A1:** Eight groups of urediniospore size (µm) in a population of 65 subsamples collected in four states in Mexico.

Group No. ^1,2^	Subsamples (Leaves No.)	State ^3^	Altitude (m)	Coffee Cultivar ^4^	Spore Size (Width ^5^ × Length ^6^; µm) ^7^
3 ^1^ a ^2^	11	Puebla	229	Caturra Rojo	29.5–40 ^5^ × 19–32.5 ^6^ (–35) ^7^
1b	1	Puebla	650	Caturra Rojo	(25–) ^7^ 28–38 × 16–25 (–28)
	2	Puebla	650	Catuai	29–36 × 18–25
	4	Puebla	650	Oro Azteca	26–36 × 18–29
	6 ^8^	Puebla	1100	Caturra Rojo	(24–) 27–37 × 16.5–25
	8 ^8^	Puebla	1100	Caturra Rojo	27–37 × 17.5–26
	9 ^8^	Puebla	1100	Costa Rica 95	(28–) 30–36 × 18–26.5
	10	Puebla	229	Caturra Rojo	(28–) 30–38 × 18–25.5
	12	Puebla	578	Caturra Rojo	27–35 (–38) × 19.5–25 (–30)
	26	Oaxaca	1054	Pluma Hidalgo	(25–) 28–36 × 18–26
	29	Oaxaca	350	Caturra Rojo	23–38 × 15–27 (–37)
	31	Oaxaca	350	Caturra Rojo	(25–) 27–38 × (16–) 18–28
2c	3	Puebla	650	Catuai	29–36 × 18–25
	5 ^8^	Puebla	1100	Pacamara	(24–) 27–37.5 × 16–27
	14 ^8^	Puebla	578	Caturra Rojo	30–37 × 17.5–25
	16	Oaxaca	773	Pluma Hidalgo	28–35.5 × 18–26
	18	Oaxaca	773	Pluma Hidalgo	23–36 × 13–26
	19	Oaxaca	905	Pacamara	(24–) 28.5–35 × (12.5–) 15–27 (–30)
	20	Oaxaca	905	Pacamara	(22–) 28–36 × (13–) 19–27
	23	Oaxaca	653	Caturra Rojo	24–36 (–38) × (14.5–) 17–26
	24	Oaxaca	560	Oro Azteca	(22–) 28–37 (–39) × (14–) 18–26 (–28)
	27	Oaxaca	1054	Pluma Hidalgo	(24–) 27–35 × (17–) 19–25 (–29)
	30	Oaxaca	350	Caturra Rojo	(20–) 25–36 (–38) × 15–28
	32	Chiapas	1241	Caturra Rojo	(21–) 29–36 (–38) × (14.5–) 19–27
	36	Chiapas	823	Caturra Rojo	26–36 × (16–) 18–26.5
	41 ^8^	Chiapas	559	Robusta	(25–) 27–36 × (15–) 19–25 (–28)
	48	Chiapas	1649	Bourbon	(23–) 26–36 × (15–) 20–26
	59	Veracruz	839	Caturra Rojo	(25–) 28–35 × 17.5–24
4d	17	Oaxaca	773	Pluma Hidalgo	25–36 (–38) × (15–) 17–25
	21	Oaxaca	653	Caturra Rojo	(22.5–) 28–34 (–36) × (14–) 17.5–25
	25	Oaxaca	560	Oro Azteca	(23.5–) 29–33.5 (–37) × (14.5–) 17–26 (–28)
	28 ^8^	Oaxaca	1054	Pacamara	28–32 (–34) × (16–) 18–25
	38	Chiapas	823	Caturra Rojo	(21–) 27–36 (–38) × 15–25 (–27)
	39	Chiapas	823	Caturra Rojo	(24.5–) 27–35 × (15–) 18–26.5
	42	Chiapas	559	Robusta	24–36 × 15–23 (–27)
	45	Chiapas	389	Caturra Rojo	(20–) 27–35 × (14–) 19–26
	53	Veracruz	1318	Maragogipe	(25–) 28–34 × (14.5–) 16–26
	58	Veracruz	1053	Caturra Rojo	(25–) 28–35 (–38) × (14.5–) 18–25
	60	Veracruz	839	Bourbon	27–35 × 17–25 (–28)
	61	Veracruz	565	Caturra Rojo	25–35 × 17.5–26
5e	35	Chiapas	1241	Caturra Rojo	(21–) 25–33.5 (–35) × (15–) 18–27 (–29)
	46	Chiapas	1649	Bourbon	(25–) 27–35 × (15.5–) 20–26 (–28.5)
	47	Chiapas	1649	Bourbon	(21–) 26–35 × (15–) 19–26 (–28)
	49	Veracruz	1318	Garnica	(22–) 27–34 × (14.5–) 16–25 (–28)
	57	Veracruz	1318	Blue Mountain	(25–) 27–34 × (15–) 17–25 (–30)
7f	13	Puebla	578	Caturra Rojo	(25.5–) 28–36 (–38) × 18–25 (–26.5)
	22	Oaxaca	653	Caturra Rojo	(24–) 28–35 × 14–25
	40	Chiapas	559	Robusta	(25–) 28–35 × 15–24
6g	7 ^8^	Puebla	1100	Caturra Rojo	26.5–34 (–37) × 16–25
	33	Chiapas	1241	Caturra Rojo	(22.5) 28–33 × 14.5–25
	34 ^8^	Chiapas	1241	Caturra Rojo	(21–) 24–32 × (15–) 17–26
	37 ^8^	Chiapas	823	Caturra Amarillo	(23.5–) 25–33.5 (–35.5) × 15–26
	43	Chiapas	389	Caturra Rojo	23–32 (–35) × (14–) 18–23 (–26)
	44	Chiapas	389	Caturra Rojo	(20–) 24–34 × (14–) 17.5–27 (–28)
	54	Veracruz	1318	Mundo Novo	(20–) 24–34 × 14–23 (–28)
	62	Veracruz	565	Typica	(20–) 28–35 × 15–24 (–28)
	63	Veracruz	1332	Colombia	(23–) 25–32 (–34) × 14–25
	64	Veracruz	1332	Oro Azteca	(21–) 24.5–35 × 14–24
	65	Veracruz	1332	Typica	24–32 (–35) × (14–) 18–25
8h	15	Oaxaca	773	Pacamara	24–32 × 15–21.5 (–26)
	50	Veracruz	1318	Garnica	(20–) 24–30.5 (–33) × 15–23 (–25.5)
	51	Veracruz	1318	Surinam	(20–) 23–34 × 15–26
	52	Veracruz	1318	Pluma Hidalgo	(19–) 25–32 × 14–21.5 (–23.5)
	55	Veracruz	1318	Moka	25–35 × 14–25
	56	Veracruz	1318	Blue Mountain	22–33 × 15–23 (–25)
Overall no.: 65 4 Overall range:			229–1649 m	17	(20–) 24–37 (–40) × (11.5–) 15–27 (–30.5) µm

^1^ Group number (No.) ordered for significant differences; ^2^ different letters indicate significant differences between groups (*p* ˂ 0.0001); ^3^ locations and climate conditions are presented in Table A2; ^4^ each different cultivar (n = 17) is highlighted in bold; ^5,6^ size range (95% interval range) [30]; ^7^ numbers in parenthesis are extreme spore width and length in µm (2.5% interval: (minimum–); (–maximum)) [30]; ^8^ sequenced samples; additionally, 7 samples (80, 95, 142, 165, 192, 211, and 234) were sequenced.

**Table A2:** Urediniospore size groups distributed per state with sampling date, location, and regional environmental conditions.

States Sampled ^1^ Groups (1–8) ^2^ Leaves Analyzed	Location (14 Regions)	Altitude (m)	Geographical Coordinates	Climate ^3^	Temperature (°C) ^4,5^		Rain ^4,5^ (mm) ^8^
					Max ^6^	Min ^7^	
Puebla ^1^: 22 and 23 August 2015							
1 ^9^ b ^10^, 3 ^11^	Mazacoatlan ^12^, Zihuateutla ^13^	1100	20.37 N, −97.86 W	Warm and humid	23	13	98
1b, 2c, 7f 5	Pozo del Tigre, Jalpan	578, 650	20.42 N, −97.87 W	Mild–warm and damp	28	13	60
3a, 1b 2	San Bartolo del Escobal, Venustiano Carranza	229	20.42 N, −97.66 W	Warm, damp, and humid	32	23	70
1b, 2c, 6g 4	Santa Rita, Xicotepec	578, 650, 823, 1100	20.27 N, −97.98 W	Mild–warm and damp	25	13	66
Oaxaca: 9 and 10 October 2015							
1b, 2c, 4d, 7f, 8h 14	San Jose del Pacifico, San Mateo	560, 653, 773, 905, 1054	15.94 N, −96.45 W	Mild–warm and humid	28 ^14^	22 ^14^	200 ^14^
1b, 2c 3	Frontera ^13^	350	15.89 N, −96.48 W	Mild–warm and humid			
Chiapas: 5 and 6 November 2015							
2c, 5e 3	Granadilla, Zinacantan	1649	16.70 N, −92.83 W	Temperate and damp	19	9	68
2c, 4d, 6g 4	La Trinidad, Union Juarez	823	15.03 N, −92.10 W	Mild–warm and humid	23	15	137
2c, 4d, 7f, 6g 6	Rosario Izapa, Tuxtla Chico	389 559	14.95 N, −92.15 W 15.00 N, −92.15 W	Warm and humid	31	21	69
2c, 5e, 6g 4	Union Juarez, Union Juarez	1241	15.89 N, −92.07 W	Mild–warm and humid	23	15	137
Veracruz: 16 and 18 November 2015							
2c 1 ^11^	Huatusco, Huatusco	839	19.17 N, −96.96 W	Warm and humid	22	12	49
4d, 5e, 6g, 8h 9	Huatusco, Huatusco	1318	19.17 N, −96.96 W	Warm and humid	22	12	49
6g 3	Ixhuatlan del Café	1332	19.05 N, −96.98 W	Temperate and humid	23	11	45
4d 1	Puerto Rico, Coatepec	1053	19.43 N, −96.90 W	Temperate and humid	23	11	80
4d, 6g 3	Tuzamapan, Coatepec	565, 839	19.20 N, −96.85 W 19.39 N, −96.86 W	Temperate and humid Regular	25	14.5	64

^1^ Four states with sampling date (chronological order); ^2^ groups classified according to urediniospore size per state and location; ^3^ for municipality; ^4^ Weather Atlas [28] (according to sampling date); ^5^ Weather spark [29]; ^6^ maximum; ^7^ minimum; ^8^ cumulative; ^9^ group number; ^10^ different letters indicate significant differences between groups (*p* ˂ 0.0001); ^11^ number of samples (total = 65 leaves); ^12^ region; ^13^ municipality; ^14^ data for the municipality.

## Appendix B

### B.1. Materials and Methods

#### Molecular Characteristics: PCR Amplification and Sequence Analysis

Among the 265 samples collected, 50 stratified samples were randomly selected based on their origin, region, altitude, and cultivar (Table A1). From an uredinium on a lesion, 10–15 urediniospores were taken using a sterile needle point under a stereoscope microscope. These urediniospores simultaneously underwent DNA extraction and PCR reaction using a Phire Plant Direct PCR Kit (Thermo Scientific^®^, Waltham, MA, USA) following the manufacturer’s directions. The ITS regions of the rRNA were amplified with the forward primer ITS5 (5′–GGAAGTAAAAGTCGTAACAAGG–3′) and the reverse primer ITS4 (5′–TCCTCCGCTTATTGATATGC–3′) [39]. Thermocycling conditions for the amplifications consisted of 5 min at 98 °C for initial denaturing, followed by 40 cycles of 5 s at 98 °C, 5 s at 63.3 °C, 20 s at 72 °C, and a final 1 min extension step at 72 °C [40]. A 1 Kb molecular marker was used for gel electrophoresis. All the amplified products were purified and sequenced in both directions by Macrogen Inc. (Seoul, South Korea). The ensuing sequences were analyzed with DNASTAR v. 5 (Lasergene, Wisconsin, MA, USA). The sequences were BLAST searched against the NCBI GenBank database using the BLASTn (Pre-formatted databases for BLAST nucleotide) tool and submitted to NCBI (https://ftp.ncbi.nlm.nih.gov/blast/db/, accessed on 18 November 2024).

### B.2. Results

#### Species Confirmation via Molecular Analysis

Sixteen subsamples were sequenced (Table A1). The electrophoresis of the PCR products corresponding to the ITS–rDNA regions revealed bands of 900–950 base pairs (bps) related to the complete ITS1 and ITS2 regions, with a portion of the 18S and 28S genes, and the complete sequence of gen 5.8S. The BLASTn search of the NCBI GenBank for the 16 sequenced subsamples (426–875 bp) showed that they all aligned with the *H. vastatrix* sequences from Colombia and Brazil, having the highest identity agreement of 99.43–100%. The 16 sequences obtained in this study were deposited in GenBank.
